# Analysis of the Relapse Rates of the Primary Closure and Limberg Flap Techniques in Pilonidal Sinus Surgery

**DOI:** 10.7759/cureus.5730

**Published:** 2019-09-23

**Authors:** Murat Kanlioz, Ugur Ekici

**Affiliations:** 1 General Surgery, Beylikdüzü Kolan Hospital, Istanbul, TUR; 2 General Surgery, İstanbul Gelisim University, Istanbul, TUR

**Keywords:** pilonidal sinus, relapse, limberg flap, primary closure

## Abstract

Background

This study aimed to assess the relapse rates at the long-term follow-up of the Limberg flap repair (LFR) and primary closure (PC) methods in the surgical treatment of pilonidal sinus disease (PSD).

Methods

The records of primary PSDs who underwent LFR and PC due to PSD were retrospectively examined. The study included patients whose surgical intervention was performed at least two years ago. The patients were contacted by phone to obtain information. They were asked whether they had a relapse or not, and their answers were recorded. The recording and analysis were ensured using the SPSS statistical program (IBM Corp, Armonk, NY, US). The groups were compared using the chi-square test. p˂0.05 was considered significant.

Results

The patients’ mean age was 23.68 ± 8.21 years, and their median age was 22 years. The overall follow-up period was 4.38 ± 2.12 years. LFR-treated patients numbered 292; 38 (13.01%) females and 254 (86.99%) males. Of the LFR-treated patients, 23 (7.87%) had a relapse, including five (13.15%) females and 18 (7.08%) males (p˂0.03). PC-treated patients numbered 184; 58 (31.52%) females and 126 (68.48%) males. Of PC-treated patients, 39 (21.19%) had a relapse, including 15 (25.86%) females and 24 (19.04%) males (p˂0.04). The relapse was more than three times higher in PC when compared to LFR (p˂0.01).

Conclusion

LFR clearly takes precedence over PC. In both methods, the relapse rate is higher in females. We believe that this is due to our tendency to be more limited in resection in women.

## Introduction

Pilonidal sinus disease (PSD) was first described by Anderson in 1847 in his article published in Boston Medical Journal [1]. However, it was Hodges who described the disease by referring to it as "PSD," which is currently used, in 1880, again in the Boston Medical Journal [2]. Since the day it was described, there have been ongoing debates on its etiopathogenesis and treatment. Following the long-term debates on whether it is acquired or inherited in terms of etiopathogenesis, there is today a consensus that it is acquired [3-4]. However, the debates on its treatment are still ongoing [5].

PSD is an infectious disease characterized by sinus opening(s) located in the sacrococcygeal region, approximately 5-6 cm from the anus. The length of the primary canal is 3-5 cm, covered inside with squamous epithelium. Except for the primary canal, other canals are shorter in length and are mostly covered with granulation tissue. The presence of hair along the canal and inside the sinus are its typical defining characteristics. The 15-30-year-old age group, during which the activation of the pilosebase glands increases, is also the period in which PSD is the most frequent. The disease occurs in women at an earlier period compared to men. Although it varies from community to community, the female-to-male ratio ranges between 1/3 and 1/5. It is more common in those with dark skin color, hairy body, oily skin, those who are overweight, and those who are exposed to prolonged sitting as well as in males [6]. Prolonged sitting and the micro-traumas that occur in the region are considered the most important factors in the etiopathogenesis. It is more prevalent in those who are exposed to prolonged sitting, especially drivers and students [7]. Since the disease was once seen in soldiers who used military vehicles on land, it was also called the jeep disease. In this study, we aimed to assess the relapse rates at the long-term follow-up of LFR and PC methods in the treatment of PSD.

## Materials and methods

Of the patients who were admitted to our clinic for the treatment of pilonidal sinus and underwent primary closure (PC) and Limberg flap repair (LFR), those with at least two years of follow-up were retrospectively examined. The study was carried out following the principles of the Helsinki Declaration. The age and gender of the patients, the method used in their treatment, the time elapsed after surgery, whether they had a relapse or not, and the treatment administered after the relapse were recorded. The patients were contacted by phone. The patients who were able to visit us were invited to the hospital and examined. Those who couldn't do so were asked whether they had a relapse or not and, if they had a relapse, whether any other treatment was administered. The study included only patients treated for primary pilonidal sinus disease. The reason why relapse cases were excluded from the study is that we intended to measure the effectiveness of both techniques in the primary disease. The data were recorded and analyzed using SPSS statistical software (IBM Corp., Armonk, NY, US). The statistical analysis of the relapse rates between the two groups was performed using the chi-square test where p˂0.05 was considered significant.

## Results

The total number of patients was 476. Ninety-six (20.16%) of whom were female and 380 (79.84%) were male. The mean age of the patients was 23.68 ± 8.21 years and their median age was 22 years. The mean age was 19.54 ± 6.38 years in females and 24.75±9.27 years whereas the median age was 19 years in females and 24 years in males (Table 1).

**Table 1:** Age statistics of patients

	Mean Age (years)	Median Age (years)
Female	19.54±6.38	19
Male	24.75±9.27	24
Overall	23.68±8.21	22

The overall follow-up period was found to be 4.38 ± 2.12 years. The follow-up period was 4.02 ± 1.96 years in the PC-treated patients and 4.47 ± 2.36 years in the LFR-treated patients (Table 2).

**Table 2:** Follow-up periods of patients

	Follow-up Period (year)
Primary Closure	4.02±1.96
Limberg Flap	4.47±2.36
Overall	4.38±2.12

Of the patients, 184 (38.65%) were treated with PC and 292 (61.35%) were treated with LFR. Of the PC-treated patients, 58 (31.52%) were females and 126 (68.48%) were males. Of the 292 LFR-treated patients, 38 (13.01%) were females and 254 (86.99%) were males. Of the 184 PC-treated patients, 39 (21.19%) had a relapse, of whom 15 (25.86%) were females and 24 (19.04%) were males. Of the 292 LFR-treated patients, 23 (7.87%) had a relapse, of whom 5 (13.15%) were females and 18 (7.08%) were males. The difference was statistically significant (p˂0.03). When the PC and LFR methods were compared, the treatment outcomes were found to be three times more successful in favor of LFR and the difference between the groups was found statistically significant (p˂0.01). In treatments with the PC method, the relapse rate was higher, to the detriment of females. Whereas the total relapse rate in the PC method was 21.19%, this rate was found to be 25.86% in females and 19.04% in males. As the relapse rates of females and males were compared, a statistically significant difference was found between the two groups (p˂0.04). The relapse rate in LFR-treated patients was 7.87%; this rate was found to be 13.15% in females and 7.08% in males (Table 3).

**Table 3:** Number of patients and the relapse rates by methods

	Limberg Flap Repair (LFR)		Primary Closure (PC)		LFR (+) PC
	Number of patients treated	Number of patients who had a relapse	Number of patients treated	Number of patients who had a relapse	Number of patients treated
Female	38(13.01%)	5(13.15%)	58(31.52%)	15(25.86%)	96(20.16%)
Male	254(86.99%)	18(7.08%)	126(68.48%)	24(19.04%)	380(79.84%)
Total	292(100%)	23(7.87%)	184(100%)	39(21.19%)	476(100%)

The relapse rates in the LFR method were found to be 1.85 times higher in females, and the difference was statistically significant (p˂0.03). The total number of relapses in both techniques was 62 (13.02%). According to the patient records, there were 25 (40.32%) patients who re-consulted us for relapse; when we contacted the remaining 37 (59.68%) patients by phone, we found that they also had a relapse.

## Discussion

We observe that the mean age and gender distribution in our study is compatible with the literature averages. Our overall follow-up period is 4.38 ± 2.12 years. We found that 62 (13.02%) out of 476 patients had a relapse during this follow-up period. When both methods are taken into consideration, 20 (20.83%) out of 96 female patients in total and 42 (11.05%) out of 380 male patients in total had a relapse. In their study, Hammet et al. reported that the relapse rates varied from 18% to 50% at the five-year follow-up although they did not indicate those values by gender [8]. However, Lopez et al. reported relapse in 10% of patients within two years following excision [9].

It is seen that women tend to prefer the PC method due to cosmetic concerns. We very often encounter limited resection demands. Therefore, the treatment is not as curative in women as it is in men. Such types of requests made by female patients are also prominent in our study. The rates of female patients in total, of those who preferred the LFR method, and of those who preferred the PC method are 20.16%, 13.01%, and 31.58%, respectively. The relapse rates in general, in LFR, and in PC were 20.83%, 13.15%, and 25.86%, respectively. We believe that the main reason why women have higher relapse rates as compared to men is that the cosmetic concerns of women restrict surgeons from ensuring a more curative treatment.

We see that while the relapse rate is 7.87% in the LFR-treated group, it was 21.19% in the PC group. In terms of relapse rates, there is almost a three-fold difference between the two techniques. In their meta-analysis, Stauffer et al. reported a relapse rate of up to 67.9% in PCs at 20 years of follow-up [10]. We see that Erkent et al. also had similar results in their study. They also reported that they had a lower relapse rate in LFR when they compared the LFR technique with the PC technique [5]. In their meta-analysis, Boshnaq et al. reported that the overall relapse was 7.7% at 18 months of follow-up. According to their results, they stated that LFR was a technique that overrides PC. They also show that the relapse rate in the PC method ranged from 4% to 37.7% [11].

Another result that we consider important is that only 25 of the 62 patients who had a relapse re-consulted us; therefore, we only had the records of those 25 patients. However, we learned that the remaining 37 patients also had a relapse when we contacted them by phone. Roughly speaking, we were aware of only 40% of our relapses. With a false positive perception, this led us to think that our relapse rates were low. Hence, once we started our study, we faced our real relapse rates. In their survey conducted on 782 surgeons, Burnett et al. reported that the surgeons are not aware of their own relapses, therefore, they believe that their relapse rates are low following PSD surgery [12].

## Conclusions

We observed that, in terms of relapse, repair with the LFR method was three times more successful when compared to the PC method in the treatment of PSD. This made us aware that limiting our surgery due to cosmetic concerns would confront us with higher relapse rates in the future. Also, this reminded us once again that if we use only the data of the patients that we follow up, we may find very low relapse rates; however, we should know that this will never reflect the truth.
